# Transcriptome Analysis of Adaptive Heat Shock Response of *Streptococcus thermophilus*

**DOI:** 10.1371/journal.pone.0025777

**Published:** 2011-10-13

**Authors:** Jin-song Li, Yun-tian Bi, Cheng Dong, Ji-feng Yang, Wan-dong Liang

**Affiliations:** 1 College of Life Science, Zhejiang Provincial Key Laboratory of Medical Genetics, Wenzhou Medical College, Wenzhou, China; 2 School of Basic Medical Science, Wenzhou Medical College, Wenzhou, China; 3 Cardiovascular Surgery, The Fourth Hospital of Harbin Medical University, Harbin, China; Baylor College of Medicine, United States of America

## Abstract

*Streptococcus thermophilus*, a gram-positive facultative anaerobe, is one of the most important lactic acid bacteria widely used in the dairy fermentation industry. In this study, we have analyzed the global transcriptional profiling of *S. thermophilus* upon temperature change. During a temperature shift from 42°C to 50°C, it is found that 196 (10.4%) genes show differential expression with 102 up-regulated and 94 down-regulated at 50°C. In particular, 1) Heat shock genes, such as *DnaK*, *GroESL* and *clpL*, are identified to be elevated at 50°C; 2) Transcriptional regulators, such as HrcA, CtsR, Fur, MarR and MerR family, are differentially expressed, indicating the complex molecular mechanisms of *S. thermophilus* adapting to heat shock; 3) Genes associated with signal transduction, cell wall genes, iron homeostasis, ABC transporters and restriction-modification system were induced; 4) A large number of the differentially expressed genes are hypothetical genes of unknown function, indicating that much remains to be investigated about the heat shock response of *S. thermophilus*. Experimental investigation of selected heat shock gene *ClpL* shows that it plays an important role in the physiology of *S. thermophilus* at high temperature and meanwhile we confirmed *ClpL* as a member of the CtsR regulon. Overall, this study has contributed to the underlying adaptive molecular mechanisms of *S. thermophilus* upon temperature change and provides a basis for future in-depth functional studies.

## Introduction

Lactic acid bacteria play curial roles in the food industry since they are massively used for the manufacture of dairy products and fermentation processes [1]. Among them, *Streptococcus thermophilus* is one of the most important bacteria in the manufacture of yogurt and is considered as the second most important industrial dairy starter after *Lactococcus lactis* [2]. Thus, illustration of adaptation mechanism of *S. thermophilus* is very important in order to optimize the choice of strains and fermentation protocols. During the past decades, despite considerable efforts worldwide by scientist and food industry, the genetic and physiological stress response of *S. thermophilus* are still far from been studied [2], [3].

Bacteria can monitor the environment and change their gene expression dynamically upon exposure to environmental factors, such as temperature, pH, osmotic activity, oxygen level and nutrient sources [4]. Among them, temperature variation is a commonly encountered environmental change in nature. To survive to various environmental stresses, bacteria have developed complex mechanisms in order to adapt to various stress tolerance [4]. It is shown that bacteria can modify the expression of about 10% of their genes in response to an increase or decrease in growth temperature [5], [6], [7], [8]. For example, to adapt to heat stress, bacteria up-regulated a number of heat shock genes, including chaperones encoded by the *DnaK* and *GroE* operons and ATP-dependent proteases (*Clp* and *Lon*). During the past decade, the molecular mechanisms underlying the heat shock responses have been investigated in many bacterial species, such as *Listeria monocytogenes* [9], *Shewanella oneidensis* [6] and *Campylobacter jejuni* [5]. Despite the importance roles of *S. thermophilus*, little is known about its potential molecular mechanism in response to temperature changes. Only recently, *S. thermophilus* has been shown to change the expression level of *ClpL* at both high and low temperature [10]. Meanwhile, the transcriptome of mixed-culture growth of *S. thermophilus* and *Lactobacillus* has been investigated [11], [12].

In order to better understand the molecular mechanisms of *S. thermophilus* adapting to environmental stresses in temperature, global transcriptional profiling of *S. thermophilus* has been investigated during cultivation at 42°C and 50°C using DNA microarray hybridization analysis. It is revealed that extensive transcriptome differences has been occurred during a temperature shift from 42°C to 50°C in *S. thermophilus.* Our study identified 196 differentially expressed genes, of which 102 are up-regulated and 94 are down-regulated. The most prominent findings were that adaptive heat shock response was characterized by the dynamic change of genes involved in heat shock genes, transcriptional regulators, signal transduction, cell wall genes, iron homeostasis, ABC transporters and restriction-modification system.

## Materials and Methods

### Bacterial strains and culture conditions

The strain used in this study was *S. thermophilus* LMG18311 (obtained from the BCCM collection, Belgium) because its genome has been completely sequenced and the organism has been used in many genetic studies [3]. *S. thermophilus* LMG18311 was cultured anaerobically at 42°C in M17 medium supplemented with 1% lactose. The bacterial growth was monitored by measuring the optical density at 600 nm (OD 600) using the spectrophotometer (Beckman DU-650).

For the heat shock experiment, a single colony was inoculated in the M17 medium at 42°C to mid-exponential phase (optical density at 600 nm OD600 of 0.2) and aliquots of the cultures were then heat shocked at 50°C at water bath for 15 min and 30 min, respectively.

### RNA extraction and hybridization

Total RNA was isolated under all experimental conditions using Trizol reagent (Invitrogen) according to the manufacturer's instructions. The amount, quality and integrity of each RNA sample were assessed spectrophotometrically and by gel analyses. The extracted RNAs were purified using the RNeasy Mini kit (QIAGEN) according to the manufacturer's protocols. Synthesis of double-stranded cDNA was carried out using the SuperScript™ Double-Stranded cDNA Synthesis Kit (Invitrogen) with 10 ug of total RNA according to the manufacturer's protocols. The labeling and hybridization were performed by the well established NimbleGen Systems (Madison, WI) described everywhere according to the NimbleGen's User's Guide for Expression Analysis [13], [14]. Oligonucleotide microarray chips the *S. thermophilus* LMG18311 were purchased from NimbleGen Systems, (Madison, WI). It contains 192,000 oligonucleotide probes (60-mer each probe) designed for all 1,889 ORFs of *S. thermophilus* LMG18311 genome with an average of each ORF of nineteen probes. In addition, each microarray chip has five technical replicates so that there were a total of 95 probes per ORF.

### Real-time quantitative RT-PCR

The microarray results were validated by the real-time quantitative RT-PCR (qRT-PCR) using the QuantiTect™ SYBR@ Green RT-PCR kit (QIAGEN) according to manufacturer's instructions. Primer Express Software v2.0 (Applied Biosystems) was used for oligonucleotide primer design. Sixteen genes (nine up-regulated and seven down-regulated) were selected for analysis. To avoid DNA contamination, each RNA sample was subjected to DNase treatment with TURBO DNase (Ambion) prior to qRT-PCR. Gene expression was tested each RNA from control and heat shock-treated samples from all three experimental replicates and the relative quantification of mRNA was performed with the ABI Prism 7300 Sequence Detection System (Applied Biosystems). RNA concentrations were as follows: 1 ug forward primer, 1 ug reverse primer and 2 ug target RNA. PCR reaction were as follows: reverse transcription at 50°C for 30 min and 95°C for 15 min, 40 cycles of denaturation at 92°C for 15 s, and extension at 55°C for 30 s. The *tuf* gene (stu0487) was used as an internal control to normalize the RNA concentration, which has a consistent expression level throughout the different heat shock microarray experiments (data not shown). The fold change of gene expression was calculated using the comparative Ct (2^−ΔΔCt^) method.

### Microarray data analysis

The slides were scanned using the Axon GenePix 4000B scanner (Molecular Devices Corporation, Sunnyvale, CA). The raw data of fluorescence intensity from the scanned images was generated using the NimbleScan program v2.5 (http://www.nimblegen.com/products/software/nimblescan/). For each transcriptional profile condition, three biological replicates were investigated and used for microarray hybridization. Meanwhile, each biological sample was performed three times in microarray hybridization. Therefore, a total of nine measurements can be obtained for each gene. Microarray data used to the differential expression analyses is the average of all nine measurements. The statistical analysis was performed using a flexible empirical Bayes model (the LNN model) in the EBArrays package in R/Bioconductor software (http://www.bioconductor.org). A gene was considered to be statistically significant if more than a 1.5 fold change was shown combined with a *P* value >0.001.

### Construction of Δ*ClpL* and Δ*CtsR* mutant

Construction of Δ*ClpL* and Δ*CtsR* mutant is similar with that of previous studies using the pG+host9 plasmid [10], [15]. In brief, firstly, two internal DNA fragments of *ClpL* and *CtsR* were amplified from *S. thermophilus* LMG18311 by PCR using specific primers (*ClpL,* forward: 5′-CTTTTCAATCAATTG ATGGG-3′ and afterward: 5′-CATTTGAGATACTGGAATACCAGTCAT-3′; *CtsR,* 5′-CAAGAAATACATCAGATAG-3′ and afterward: 5′-GATAGC CGCATCTTCGCCTAAAAC-3′). The PCR products were purified using PCR purification Kit (QIAGEN) and inserted into the pG+host9 plasmid. Then, the resulting pG+host9 was electroporated into *S. thermophilus* LMG18311 and the transformed cells were incubated in anaerobic condition with 0.4 µg/mL erythromycin at 30 °C for 4 h and followed by 48 h at 42 °C. Finally, cells were diluted and plated on LM17 plates containing 0.4 µg/mL erythromycin and incubated in anaerobic condition at 42°C. Integrants was screened as erythromycin resistance and correct insertion of target gene fragments was checked by colony PCR analysis using specific primers (*ClpL,* forward: 5′-GGGTTTTGGTCAGTAAAAGTCAA-3′ and afterward: 5′-CGTAGCACCCAAGGAAAGTC-3′; *CtsR,* 5′-GTCAAATTAGACTGGAGG-3′ and afterward: 5′-AATGGCCTGCATTTTTCTTG-3′).

## Results and Discussion

### Data validation and classification of heat shock related genes

*S. thermophilus*, a gram-positive facultative anaerobe, is an industrially important bacteria widely used in dairy fermentation [2]. To gain novel insights into which of the organism's genes involved in temperature adaptations, we used DNA arrays to compare the transcriptional profiles of *S. thermophilus* upon a temperature shift from 42°C to 50°C at 15 min and 30 min. A minimum threshold of 1.5 folds change of gene expression level and a *P* value of smaller than 0.001 was used as the cutoff values. Under these conditions, in total, 196 genes show significantly differential expression at 50°C: 180 genes (89 induced and 91 repressed) at 15 min and 179 genes (95 induced and 84 repressed) at 30 min (Table S1, Table 1).

**Table 1:** The top seventy differentially expressed genes during a temperature shift from 42°C to 50°C at 15 min and 30 min in *S. thermophilus.*

Gene ID	COGs	Gene	Fold Change at 50°C		Description
			15 min	30 min	
stu0120	O	*DnaK*	17.3	19.8	molecular chaperone
stu0119	O	*GrpE*	14.5	17.6	heat shock protein
stu0121	O	*DnaJ*	10.5	11.6	chaperone protein
stu0203	O	*GroES*	8.1	10.5	co-chaperonin
stu0204	O	*GroEL*	5.4	5.3	chaperonin
stu1614	O	*ClpL*	3.2	2.7	ATP-dependent Clp protease
stu0356	O	*clpP*	1.9	3.2	ATP-dependent Clp protease
stu0705	V	*hsdR1*	4.5	4.6	type I restriction-modification system
stu0317	TK	*CovR*	1.8	4.2	response regulator
stu1802	R	*-*	7.6	4.6	hypothetical protein
stu1875	R	*-*	2.8	4.3	hypothetical protein
stu1666	R	*-*	−2.7	−3.3	putative ABC transporter ATP binding protein
stu1665	R	*-*	−3.3	−2.1	putative ABC transporter permease protein
stu1615	R	*-*	−3.5	−3.7	hypothetical protein
stu1005	P	*pstB2*	5.3	5.6	phosphate transport system ATP-binding protein
stu1002	P	*pstC1*	4.5	3.2	phosphate transport system permease protein
stu1003	P	*pstC2*	3.8	2	phosphate transport system permease protein
stu0607	P	*feoA*	2.7	3.5	ferrous iron uptake transporter protein A
stu1025	P	*fatB*	2.4	3.1	iron complex transport substrate-binding protein
stu0724	P	*Fur*	−3	−3.4	transcriptional regulator
stu0569	M	*-*	4.5	4.3	glycosyl transferase family protein
stu0762	M	*dltB*	−2.8	−3.8	integral membrane protein
stu1465	L	*radC*	3.1	2.5	DNA repair protein
stu0118	K	*hrcA*	6.8	4.6	heat-inducible transcription repressor
stu0065	K	*MarR*	3.8	5.2	transcriptional regulator
stu0432	K	*MarR*	1.8	3.1	transcriptional regulator
stu1600	K	*MerR*	−1.5	−4.3	transcriptional regulator
stu0931	K	*TetR*	−1.6	3.3	transcriptional regulator
stu1868	K	*rpoB*	−3.8	−3.4	DNA-directed RNA polymerase
stu0076	K	*CtsR*	−4.3	−4.8	transcriptional regulator
stu0419	J	*-*	5.4	4.5	acetyltransferase
stu0094	J	*rpsI*	4.2	2.3	30S ribosomal protein S9
stu0074	J	*tsf*	3.6	3.1	elongation factor Ts
stu0451	J	*metG*	3.2	4.3	methionyl-tRNA synthetase
stu1808	J	*rpmH*	3.1	2.8	50S ribosomal protein L34
stu1932	J	*rplW*	−2.6	3.6	50S ribosomal protein L23
stu1926	J	*rpmC*	−3.3	−3.6	50S ribosomal protein L29
stu0151	J	*def*	−3.4	−3.5	peptide deformylase
stu1814	J	*gltX*	−3.6	−3.1	glutamyl-tRNA synthetase
stu0154	J	*rpsO*	−5.3	−5	30S ribosomal protein S15
stu1196	G	*pyk*	−1.8	−4.2	pyruvate kinase
stu0807	F	*cdd*	2.4	3.8	cytidine deaminase
stu1316	E	*sdaB*	4.9	2.5	L-serine dehydratase beta subunit
stu1878	E	*thrC*	3.4	3	threonine synthase
stu0363	E	*livF*	1.8	3.8	branched-chain amino acid transport system ATP-binding protein
stu0159	E	*-*	−3.6	−3.1	polar amino acid transport substrate-binding protein
stu1461	E	*nifS3*	−5	−3.5	Putative cysteine desulfurase
stu0479	C	*atpB*	−3.2	−3.3	F0F1 ATP synthase
stu0478	C	*atpE*	−6.3	−4.8	F0F1 ATP synthase
stu0863	*-*	*-*	4	3.5	hypothetical protein
stu1575	*-*	*-*	4	1.6	hypothetical protein
stu1378	*-*	*-*	3.6	2	hypothetical protein
stu0087	*-*	*-*	3	1.6	hypothetical protein
stu0829	*-*	*-*	2.8	3.3	hypothetical protein
stu1722	*-*	*-*	2.8	3.4	hypothetical protein
stu0161	*-*	*-*	2.4	3.6	hypothetical protein
stu1075	*-*	*-*	1.4	3.7	hypothetical protein
stu0013	*-*	*-*	−3.2	−3.7	hypothetical protein
stu0135	*-*	*-*	−3.4	−5.4	hypothetical protein
stu1454	*-*	*-*	−3.5	−1.6	hypothetical protein
stu1533	*-*	*-*	−3.6	−3	hypothetical protein
stu1639	*-*	*-*	−3.9	−1.9	hypothetical protein
stu1047	*-*	*-*	−4.2	−3.6	hypothetical protein
stu1459	S	*-*	3.6	2	hypothetical protein
stu0440	S	*-*	2.8	4.6	hypothetical protein
stu1248	S	*-*	1.8	3.5	hypothetical protein
stu0423	S	*-*	−3.3	−2.7	hypothetical protein
stu1253	S	*-*	−4.1	−3.9	hypothetical protein
stu0888	S	*-*	−8	−5.6	hypothetical protein
stu0868	S	*-*	−9.3	−5.4	hypothetical protein

**Figure 1:**
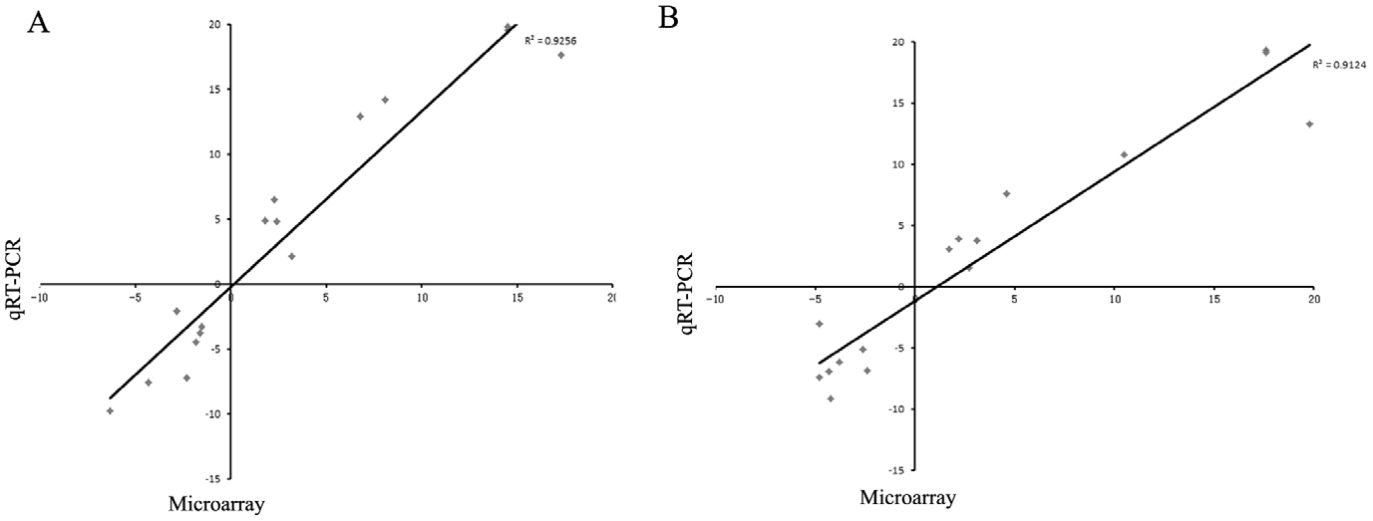
Validation of microarray differentially expressed genes by qRT-PCR. (A) Correlation of microarray results with qRT-PCR at 50°C at 15 min. (B) Correlation of microarray results with qRT-PCR at 50°C at 30 min.

To validate the level of reliability of the microarray results, sixteen differential expression genes representing a wide range of fold change values were selected for quantitative RT-PCR experiments with the same RNA samples used in the array hybridizations. Among them, nine genes were over-expressed during heat shock (*GrpE*, *DnaK*, *GrpE*, *GroES*, *hrcA*, *hsdS1*, *ClpL*, *livH* and *fatB*) and seven genes were repressed (*dltB*, *pyk*, *atpE*, *dltD*, *dltC*, *CtsR* and *MerR*). In general, it is shown that it is a coincidence in the gene expression level identified by both methods, indicating a good correlation between microarray hybridization and quantitative RT-PCR data (Figure 1). However, the fold change observed with microarray were usually lower to those observed with quantitative RT-PCR, which has been reported previously [5], [6]. This result may be due to the detection limit of microarrays and the complex normalization strategies that are used prior to the analysis.

**Figure 2:**
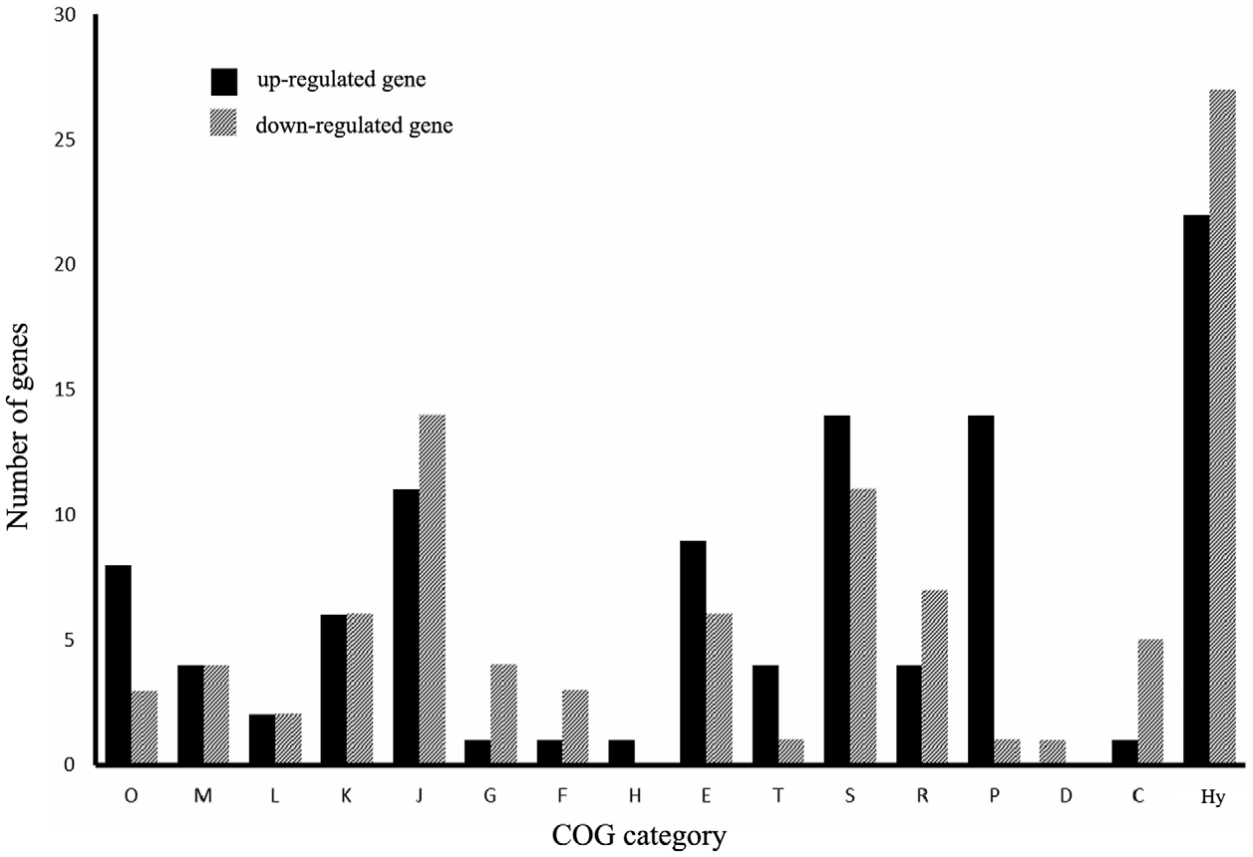
Differentially expressed genes grouped by COG functional classification. Columns: O: posttranslational modification, protein turnover, chaperones; M: cell envelope biogenesis, outer membrane; L: DNA replication, recombination and repair; K: transcription; J: translation, ribosomal structure and biogenesis; G: carbohydrate transport and metabolism; F: nucleotide transport and metabolism; H: coenzyme metabolism; E: amino acid transport and metabolism; T: signal transduction mechanisms; S: function unknown; R: general function prediction only; P: inorganic ion transport and metabolism; D: cell division and chromosome partitioning; C: energy production and conversion and Hy: hypothetical protein.

To illustrate the differentially-regulated genes more thoroughly, we categorized them based on the Clusters of Orthologous Groups (COG) category. It is shown that the pattern and distribution of differentially expressed genes differs for different COG categories (Figure 2). As expected, a large number of differentially-regulated genes were involved in translation, ribosomal structure and biogenesis (COG J). As expected, a large number of these most differential expressed genes at ribosomal proteins (Table 1). The repression of expression of ribosomal proteins at 50°C is consistent with the slower growth of *S. thermophilus* cells during the high temperature condition.

### Induction of heat shock proteins

**Figure 3:**
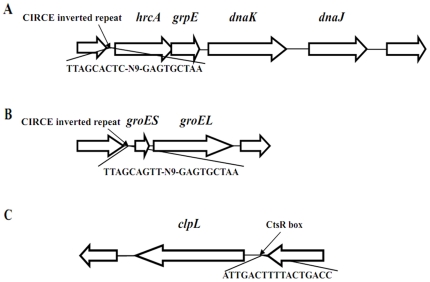
Gene organization of three gene clusters identified during a temperature shift from 42°C to 50°C in *S. thermophilus*. (A): The *DnaK* operon with the CIRCE motif. (B): The *GroESL* operon with the CIRCE motif. (C): The *clpL* gene with the CtsR box.

Transcriptome data obtained after exposure of *S. thermophilus* to heat shock showed that genes encoding proteins involved COG category O (posttranslational modification, protein turn-over and chaperones) were significantly differential expressed. Among heat shock genes, the *DnaK* (stu0120), a member of the hsp70 gene family, was identified as the most significantly up-regulated gene, the expression level of which was elevated by 17.3 folds at 15 min and 19.8 folds at 30 min upon temperature change from 42°C to 50°C, respectively. The other components of the *DnaK* operon (http://www.microbesonline.org/operons/gnc264199.html) were highly responded simultaneously, which includes the major molecular chaperones *GrpE* (stu0119), *DnaJ* (stu0121) and heat-inducible transcription repressor hrcA (stu0118). It is indicated that the classical chaperone *DnaK* that is negatively regulated by the transcriptional regulator hrcA which binds to the CIRCE motif [16]. We found that the *DnaK* operon is belonging to this classical chaperone in *S. thermophilus*, which has upstream CIRCE inverted repeats (TTAGCACTC-N9-GAGTGCTAA) essential for hrcA binding to (Figure 3A). However, the sequence of the *S. thermophilus DnaK* CIRCE region (TTAGCACTC-N9-GAGTGCTAA) is different from the CIRCE consensus sequence (TTGGCGCTC-N9-GAGTGCTAA) with two of the mismatches in the first repeat [16]. The organization of *HrcA* with the *GrpE*, *DnaK* and *DnaJ* into a single operon may facilitate *S. thermophilus* to respond to temperature changes easily and quickly.

In addition, we found that the *GroESL* operon consisting of *GroES* (stu0203) and *GroEL* (stu0204) were also over-expressed following temperature upshifts from 42°C to 50°C (Figure 3B). *GroESL* operon is documented to play important roles in the folding and stability of some proteins in response to various environmental stresses, including heat shock, pH and oxidative stress [17]. Searching the upstream of GroESL operon shows that it also has a CIRCE motif sequence (TTAGCAGTT-N9-GAGTGCTAA). This observation along with the up-regulation of *HrcA* (*HrcA* exerts its activity through binding to CIRCE) upon heat shock indicated that *HrcA* might involved in negative regulation of *GroESL* operon in *S. thermophilus* just as it does in *B. subtilis* [16]. Interestingly, the genome of S. thermophilus LMG18311 was found to contain only a *HrcA* (stu0118) in the *DnaK* operon and only two CIRCE motifs mentioned above. Therefore, it is suggested that the *DnaK* and *GroESL* operon are negatively regulated at transcription level by the single HrcA repressor in interacting with its operator, the CIRCE motif.

Other heat shock-related genes, *ClpL* (stu1614), *htpX* (stu0715), *parC* (stu0589) and *clpP* (stu0356) were also up regulated in *S. thermophilus* under heat stress. These genes are characterized to assist protein folding and remove of protein aggregates. It is possible that they are needed to be over expressed to help to exclude unwanted proteins and correct proper protein folding newly synthesized to adapt metabolism process during the new environment.

### Induction of transcriptional regulators

Genomic analysis of *S. thermophilus* LMG18311 revealed that it encodes a number of 71 transcriptional regulators (http://transcriptionfactor.org), which consists of about 3.7% of total encoded genes. It is found that many transcriptional regulators encoded in *S. thermophilus* were expressed at similar levels at 42°C and 50°C. However, interestingly, many transcriptional regulators showed significant expression changes at 50°C, including Fur, MarR, and MerR family beside HrcA, CtsR and response regulators of two-component regulatory system. In particular, a Fur regulator (stu0724) and the iron chelator dpr (stu0723) were more than 3 folds and 2 folds down-regulated at 50°C, respectively. This observation is in accordance with previous study that temperature change can influence the differential expression of genes related to iron homeostasis proteins [7]. Fur is a global regulator iron ion transport, which uses Fe^2+^ as a cofactor to bind to promoter (Fur box) of genes involved in iron acquisition processes and represses the transcription [18]. Consistent with the down-regulation of Fur, the iron complex ABC transporter, consisting of *fatA* (stu1026), *fatB* (stu1025), *fatC* (stu1027) and *fatD* (stu1028), was up-regulated at 50°C in *S. thermophilus*. In addition, a second operon *feoAB* (stu0607 and stu0608) involved in iron uptake and transport is also up-regulated 2.7 to 2.9 at 40 uC in *S. thermophilus*. Further investigation of upstream of *fatABCD* and *feoAB* revealed the appearance of a putative Fur box palindromic for Fur.

Two transcriptional regulators (stu0065 and stu0432) belonging to MarR family was up-regulated more than 3.5 folds and 1.8 folds at 50°C, respectively. Previous studies show that the MarR family transcriptional regulators were also differently expressed in and *Thermothoga maritime* and *Exiguobacterium sibiricum* under high temperatures [8], [19]. Thus, it is indicated that the MarR family may play roles in the adaptive molecular mechanisms upon temperature change. Meanwhile, a homology of MerR families of transcriptional regulator (stu1600) was down-regulated about 1.5 folds at 15 min upon temperature change from 42°C to 50°C. However, it exhibits lower expression level with a 4.3 folds at 30 min at 50°C. Although little is known about the functional role of stu1600, MerR family, a group of transcriptional activators, has been shown to respond to the presence of essential and toxic metals [20].

Additionally, two transcriptional regulators responded in opposite ways at 15 min and 30 min upon high temperature at 50°C. Expression of the transcriptional regulator stu0452 (belongs to LysR family) was increased about 2.7 folds at 15 min at 50°C, whereas it subsequently decreased about 1.9 folds 30 min at 50°C. The transcriptional regulator stu0931, a putative positive regulator belonging TetR family, was 1.6 folds down-regulated after 10 min heat shock at 50°C, but 3.3 folds up-regulated at 30 min relative to 10 min. The results suggest that positive regulator LytSR and TetR may hold different mechanisms to regulate gene expression to cope with heat stress under different heat shock conditions.

Taken together, the significantly differential expression of many *S. thermophilus* transcriptional regulators illustrates the complex molecular mechanisms of *S. thermophilus* adapting to heat shock. However, how these transcriptional regulators respond to heat shock is poorly characterized and further studies need to address this issue.

### Induction of signal transduction and membrane proteins

Signal transduction has been indicated to be one of main mechanisms for bacteria to adapt to various environmental changes. One of these mechanisms involves two-component regulatory systems, consisting of a histidine kinase and a response regulator, by which bacteria can to sense and respond to changes in many different environmental conditions [21]. The histidine kinase receives specific signals and transmits them to a partner response regulator via phosphorelay. Genomic analysis of *S. thermophilus* LMG18311 revealed it encodes 19 two-component proteins (http://genomics.ornl.gov/mist/view_organism.php?organism_id=267). Among them, we found that two operons are differently expressed during a temperature shift from 42°C to 50°C. The first operon *covRS*, consisting of a histidine kinase (CovS, stu0318) and a response regulator (*CovR*, stu0317) are significantly up-regulated at 50°C. In particular, *CovS* was 2 folds elevated at 50°C relative to 42°C at both 15 min and 30 min. While, *CovR* was elevated at 50°C by 1.8 folds and 4.2 folds at 15 min and 30 min, respectively. It is suggested that the *covRS* system may be also involved in general heat shock response since their up-regulation was also observed in other *Streptococcus* species [7]. The second operon, consisting of a histidine kinase (stu1421) and a response regulator (stu1420), is up-regulated about 2 folds at 50°C. The homolog of this operon in *Lactococcus lactis* subsp. cremoris MG1363 is proposed to be involved in response to salt or osmotic stress [22]. In addition, we found that a response regulator stu1380, belonging to the transcriptional regulator of OmpR family, was down-regulated both the 15 min and 30 min upon temperature change from 42°C to 50°C. However, unfortunately, its functional role is poorly characterized in *Streptococcus*.

The bacterial cell wall is a very complex structure, which provides structural integrity to the cell and has primary protection function against environmental stresses. Currently, however, little is known about the cell wall of *S. thermophilus*. Under the heat shock progress of *S. thermophilus*, an interesting observation was that several cell envelope and membrane biogenesis were differentially expressed. For example, two glycosyl transferase (stu0569 and stu0570) involved in the synthesis of cell wall carbohydrate (cellulose), have been up-regulated during a temperature shift from 42°C to 50°C. Additionally, an operon, consisting of *dltB* (stu0762, D-alanine export protein), *dltC* (stu0763, D-alanine ligase) and *dltD* (stu0764, D-alanine transfer protein), has been down-regulated with about 1.6–3.8 folds changes, which is responsible for D-alanine esterification of lipoteichoic acids and play important roles in cell wall synthesis [23]. *murD* (stu0731) and *murG* (stu0732) encoding key enzymes for the peptidoglycan synthesis are also up-regulated upon temperature change from 42°C to 52°C. Meanwhile, a putative lipoprotein (stu1024) and two putative membrane proteins (stu1993 and stu1996) involved in cell wall/membrane biogenesis have been differently expressed under heat shock. These observations indicated that formation and restructuring of the cell wall has been essentially to prevent cell lysis during heat shock process in *S. thermophilus*. The significantly differential expression of differentially expressed suggesting *S. thermophilus* may dynamically change its cell membrane composition in order to adapt to high temperature stress.

### Miscellaneous observations

Several genes related to ABC transporters are differentially expressed at 50°C. stu0158 (ATP-binding protein) and stu0159 (substrate binding protein), forming an operon involved in polar amino acid transport, are significantly down-regulated more than 2 and 3 folds at 50°C, respectively. The three genes associated with branched-chain amino acid transporter, consisting of *livJ* (stu0359), *livH* (stu0360) and *livG* (stu0362), are over-expressed at 50°C at 15 min. The other two genes of this operon, *livM* (stu0361) and *livF* (stu0363), are over-expressed at 50°C at 30 min. In bacteria, two different phosphate transport systems have been identified: phosphate inorganic transport system (low affinity Pit) and phosphate specific transport system (*Pst*). The phosphate specific *Pst* uptake system consists of five members: a substrate binding protein, two permease proteins with six membrane spanning helices and two ATP binding proteins [24]. In *S. thermophilus*, the *pstSCB-phoU* operon, consisting of *pstS* (stu1001), *pstC1* (stu1002), *pstC2* (stu1003), *pstB1* (stu1004), *pstB2* (stu1005) and *phoU* (stu1006), has higher expression level upon temperature change to 50°C. Therefore, it is speculated that amino acid and phosphate transport may be enhanced during heat shock response.

Interestingly, three genes related to type I restriction-modification system are significantly differential expression during a temperature shift from 42°C to 50°C. They are *hsdR1* (stu0705, R subunit), *hsdS1* (stu0708, S subunit) and *hsdM1* (stu0711, M subunit). The functional role of type I restriction-modification system remains unknown in *S. thermophilus*. The general function of the restriction-modification system implicates in cellular defense and represents a barrier against foreign DNA through destroying them [25]. Thus, the up-regulation expression of restriction-modification enzyme may allow *S. thermophilus* to protect bacteria from invading foreign DNA, such as bacteriophage genomes.

Furthermore, several genes display differential expression involved in COG category of energy production and conversion (COG C) (Figure 2). Representative download-regulated genes involved in energy metabolism encode for three ATP synthases (stu0478, stu0479 and stu0485), a ferrodoxin-like protein (stu1137), a pyruvate kinase (stu1196), a pyridine nucleotide-disulfide oxidoreductase (stu0557) and a glycerol uptake facilitator protein (stu1671). These results suggest that the decreased of energy metabolism is consistent with slower growth of *S. thermophilus* during high temperature.

### Induction of genes with unknown function

Among the significantly differential expressed genes, we observed that a large percentage of (74 genes, 38%) both up- and down-regulated genes belong to the functional category of function unknown (COG S) and hypothetical proteins (Figure 2 and Table S1). Although with unknown function, they may have novel physiological roles associated with the process of adaptive temperature regulation. In the view of sequence length, the majority of these genes encode proteins with amino acid sequence length shorter than 100 aa. In the view of operon organization (http://www.microbesonline.org/operons/gnc264199.html), we found that the majority of these genes belong to the operon with only a single gene. However, five operons with more than two genes have also been observed. To shed more lights of these multiple gene operons to be differentially expressed, they are Blastp against protein deposited in the IMG database (http://img.jgi.doe.gov/cgi-bin/pub/main.cgi). Potential ortholog was considered if both have a two-way best hit with each other and shows a sequence similarity greater than 35%. This in-depth investigation has identified two candidate operons with conserved orthologous gene in other organisms. In particular, genes comprising the stu0868 and stu0888 operon exhibited the highest expression level among the differentially expressed genes with unknown function. The stu0888 is predicted to be a transmembrane protein with nine transmembrane helices (http://www.cbs.dtu.dk/services/TMHMM/) and conserved across many distant bacterial species. In addition, an operon consists of four hypothetical genes (stu0830, stu0831, stu0832 and stu0833) are increased with the expression level under the heat shock response. Among them, stu0830 is predicted to be a transmembrane protein with two transmembrane helices. These candidate unknown function genes to be significant differential expressed can serve as a basis for further physiological role investigation in *S. thermophilus*.

### Important role of *ClpL* for heat shock response and negatively regulated by *CtsR*

**Figure 4:**
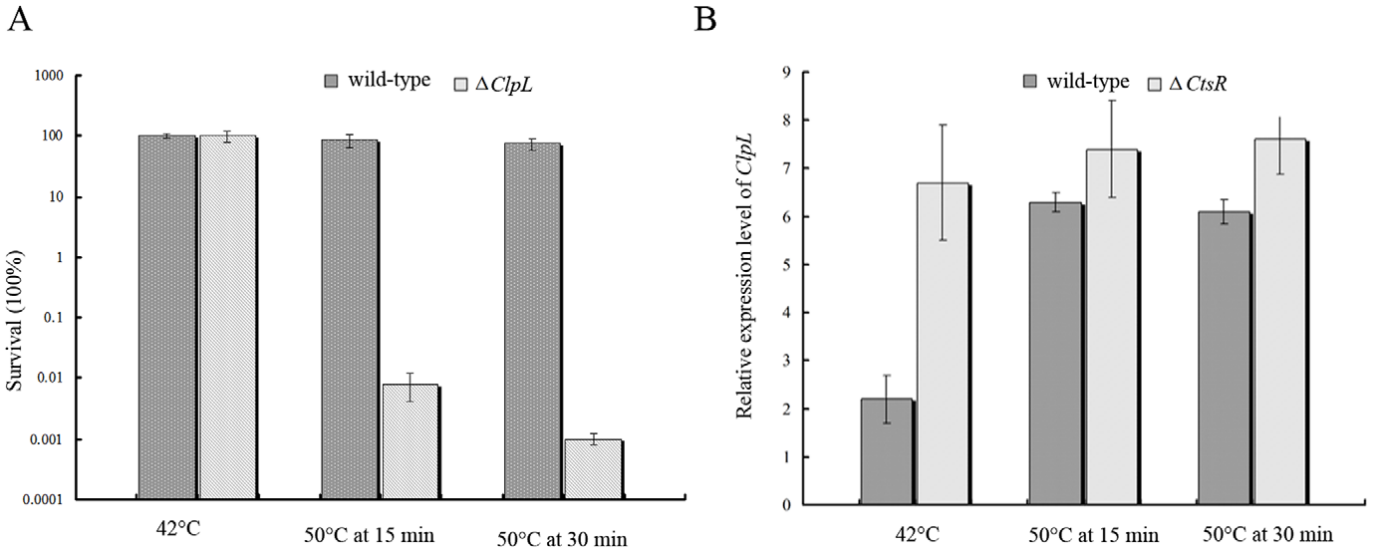
The important role of *ClpL* for heat shock response and regulated by transcriptional regulator *CtsR*. (A). Survival of wild-type *S. thermophilus* LMG18311 and Δ*ClpL* mutant at 42°C and 50°C. (B). Expression profile of *ClpL* from wild-type *S. thermophilus* LMG18311 and Δ*CtsR* mutant at 42°C and 50°C. All the results were obtained from three independent experiments and the data are represented as means±SD.

Among the differentially expressed heat shock genes, it is found that, a *ClpL* gene (stu1614), belonging to the Hsp100 family of Clp ATPases involved in stress response, was up-regulated about 3 folds at 50°C (Table 1). In *S. thermophilus* SFi39, it is indicated that the expression of *ClpL* was induced by both heat and cold shocks [10]. Such phenomenon can also be detected in several bacteria, such as *S. pneumoniae* [26] and *S. aureus* [27], suggesting that *ClpL* is a crucial part of heat shock responses in *S. thermophilus*. In order to further characterize the function of *ClpL* in *S. thermophilus*, we experimentally investigated the survival status of *S. thermophilus* in the form of wild-type and Δ*ClpL* mutant at 42°C and 50°C (Figure 4A). It is shown that both kinds of cells exhibited similar profiles of growth at 42°C. However, after upon temperature upshift at 50°C, the growth status of Δ*ClpL* mutant cells was significantly decreased and less tolerant to heat-shock in comparison with that of wild-type cells. Such observation indicated that expression of *ClpL* upon heat shock was important for *S. thermophilus* to survive at high temperature environment.

In several *Lactobacillus* species closely related to *S. thermophilus*, such as *Lactobacillus gasseri*, it is shown that the expression of *ClpL* is regulated by *HrcA* [28]. However, searching the upstream of *ClpL* has not detected any CIRCE motif sequence in *S. thermophilus*, suggesting the expression regulation of *ClpL* in *S. thermophilus* may be different from that of *Lactobacillus* species. In contrast, searching the upstream of *ClpL* has identified a CtsR box (consensus sequence, RGTCADNNANRGTCADN) with the sequence of ATTGACTTTTACTGACC in *S. thermophilus* LMG18311 (Figure 3C). Interestingly, we found that a negative transcriptional regulator *CtsR* (stu0076) is down-regulated upon temperature upshift at 50°C. Such observation suggests that expression of *ClpL* may be negatively regulated by the CtsR regulator in *S. thermophilus* in comparison with that of independence to CtsR in several closely related *Lactobacillus* species. To experimentally validate the role of CtsR in regulation of expression of *ClpL*, Δ*CtsR* mutant strain of *S. thermophilus* LMG18311 was constructed. In comparison with wild-type *S. thermophilus* LMG18311 at 42°C, an over-expression of about 3 folds of *ClpL* has been shown in Δ*CtsR* cells according to the real-time quantitative RT-PCR (Figure 4B). Similar expression profile can be also observed for the Δ*CtsR* cells at 50°C 15 min and 30 min. These experimental results confirmed that *ClpL* is a member of the CtsR regulon and negatively regulated by *CtsR*.

### Conclusions

In conclusion, this study has used transcriptional profiling strategy to genome-wide investigate adaptive heat shock response of *S. thermophilus*. Transcriptional profiles indicated that heat shock has a great impact on the gene expression of *S. thermophilus*. Subsequently, a number of 196 genes have been identified to show differential expression upon temperature change of 42°C to 50°C. The most important themes obtained from this study were that: 1) Heat shock genes, such as *DnaK*, *GroESL* and *clpL*, are shown to be up-regulated at upon temperature upshift to 50°C; 2) Many transcriptional regulators, such as HrcA, CtsR, Fur, MarR and MerR family, are differentially expressed; 3) Many genes encoding proteins involved in signal transduction, cell wall genes, iron homeostasis, ABC transporter and restriction-modification system were differentially regulated in response to heat shock; 4) Many differentially regulated genes are hypothetical genes of unknown function in response to temperature change, suggesting much remains to be investigated about the heat shock response of *S. thermophilus*. This study shed considerable insights into the better understanding of dynamically genetic changes occurred in response to heat shock in *S. thermophilus*. The candidate genes to be significant differential expressed can serve as a basis for further functional role investigation in *S. thermophilus*.

## Supporting Information

Table S1 **The differentially expressed genes during a temperature shift from 42°C to 50°C at 15 min and 30 min in** ***S. thermophilus.***
(DOC)

## References

[1] Novak L, Cocaign-Bousquet M, Lindley ND, Loubiere P (1997). Metabolism and Energetics of Lactococcus lactis during Growth in Complex or Synthetic Media.. Appl Environ Microbiol.

[2] Hols P, Hancy F, Fontaine L, Grossiord B, Prozzi D (2005). New insights in the molecular biology and physiology of Streptococcus thermophilus revealed by comparative genomics.. FEMS Microbiol Rev.

[3] Bolotin A, Quinquis B, Renault P, Sorokin A, Ehrlich SD (2004). Complete sequence and comparative genome analysis of the dairy bacterium Streptococcus thermophilus.. Nat Biotechnol.

[4] Ferenci T, Spira B (2007). Variation in stress responses within a bacterial species and the indirect costs of stress resistance.. Ann N Y Acad Sci.

[5] Stintzi A (2003). Gene expression profile of Campylobacter jejuni in response to growth temperature variation.. J Bacteriol.

[6] Gao H, Wang Y, Liu X, Yan T, Wu L (2004). Global transcriptome analysis of the heat shock response of Shewanella oneidensis.. J Bacteriol.

[7] Mereghetti L, Sitkiewicz I, Green NM, Musser JM (2008). Remodeling of the Streptococcus agalactiae transcriptome in response to growth temperature.. PLoS One.

[8] Pysz MA, Ward DE, Shockley KR, Montero CI, Conners SB (2004). Transcriptional analysis of dynamic heat-shock response by the hyperthermophilic bacterium Thermotoga maritima.. Extremophiles.

[9] Azizoglu RO, Osborne J, Wilson S, Kathariou S (2009). Role of growth temperature in freeze-thaw tolerance of Listeria spp.. Appl Environ Microbiol.

[10] Varcamonti M, Arsenijevic S, Martirani L, Fusco D, Naclerio G (2006). Expression of the heat shock gene clpL of Streptococcus thermophilus is induced by both heat and cold shock.. Microb Cell Fact.

[11] Herve-Jimenez L, Guillouard I, Guedon E, Boudebbouze S, Hols P (2009). Postgenomic analysis of streptococcus thermophilus cocultivated in milk with Lactobacillus delbrueckii subsp. bulgaricus: involvement of nitrogen, purine, and iron metabolism.. Appl Environ Microbiol.

[12] Sieuwerts S, Molenaar D, van Hijum SA, Beerthuyzen M, Stevens MJ (2010). Mixed-culture transcriptome analysis reveals the molecular basis of mixed-culture growth in Streptococcus thermophilus and Lactobacillus bulgaricus.. Appl Environ Microbiol.

[13] Snyder JA, Haugen BJ, Buckles EL, Lockatell CV, Johnson DE (2004). Transcriptome of uropathogenic Escherichia coli during urinary tract infection.. Infect Immun.

[14] Ward SK, Hoye EA, Talaat AM (2008). The global responses of Mycobacterium tuberculosis to physiological levels of copper.. J Bacteriol.

[15] Zotta T, Asterinou K, Rossano R, Ricciardi A, Varcamonti M (2009). Effect of inactivation of stress response regulators on the growth and survival of Streptococcus thermophilus Sfi39.. Int J Food Microbiol.

[16] Zuber U, Schumann W (1994). CIRCE, a novel heat shock element involved in regulation of heat shock operon dnaK of Bacillus subtilis.. J Bacteriol.

[17] Zeilstra-Ryalls J, Fayet O, Georgopoulos C (1991). The universally conserved GroE (Hsp60) chaperonins.. Annu Rev Microbiol.

[18] Hantke K (2001). Iron and metal regulation in bacteria.. Curr Opin Microbiol.

[19] Rodrigues DF, Ivanova N, He Z, Huebner M, Zhou J (2008). Architecture of thermal adaptation in an Exiguobacterium sibiricum strain isolated from 3 million year old permafrost: a genome and transcriptome approach.. BMC Genomics.

[20] Brown NL, Stoyanov JV, Kidd SP, Hobman JL (2003). The MerR family of transcriptional regulators.. FEMS Microbiol Rev.

[21] Stock AM, Robinson VL, Goudreau PN (2000). Two-component signal transduction.. Annu Rev Biochem.

[22] O'Connell-Motherway M, van Sinderen D, Morel-Deville F, Fitzgerald GF, Ehrlich SD (2000). Six putative two-component regulatory systems isolated from Lactococcus lactis subsp. cremoris MG1363.. Microbiology.

[23] Neuhaus FC, Baddiley J (2003). A continuum of anionic charge: structures and functions of D-alanyl-teichoic acids in gram-positive bacteria.. Microbiol Mol Biol Rev.

[24] van Veen HW (1997). Phosphate transport in prokaryotes: molecules, mediators and mechanisms.. Antonie Van Leeuwenhoek.

[25] Wilson GG, Murray NE (1991). Restriction and modification systems.. Annu Rev Genet.

[26] Kwon HY, Kim SW, Choi MH, Ogunniyi AD, Paton JC (2003). Effect of heat shock and mutations in ClpL and ClpP on virulence gene expression in Streptococcus pneumoniae.. Infect Immun.

[27] Frees D, Chastanet A, Qazi S, Sorensen K, Hill P (2004). Clp ATPases are required for stress tolerance, intracellular replication and biofilm formation in Staphylococcus aureus.. Mol Microbiol.

[28] Suokko A, Poutanen M, Savijoki K, Kalkkinen N, Varmanen P (2008). ClpL is essential for induction of thermotolerance and is potentially part of the HrcA regulon in Lactobacillus gasseri.. Proteomics.

