# Supplementary material for: Transcriptome Analysis of Adaptive Heat Shock Response of Streptococcus thermophilus
Source: PLoS One. 2011 Oct 13;6(10):e25777. doi: 10.1371/journal.pone.0025777 (PMC3192767; doi:10.1371/journal.pone.0025777)
Supplement: Table S1 — The differentially expressed genes during a temperature shift from 42°C to 50°C at 15 min and 30 min in S. thermophilus. (DOC) [file pone.0025777.s001.doc]

**Table S1. The differentially expressed genes during a temperature shift from 42°C to 50°C at 15 min and 30 min in *S. thermophilus***

| **Gene ID** | **COGs** | **Gene** | **Fold Change at 50°C** | | **Description** |
| --- | --- | --- | --- | --- | --- |
|  |  |  | **15 min** | **30 min** |  |
| stu0012 | O | *ftsH* | -2.3 | -2.8 | cell division protein |
| stu0119 | O | *GrpE* | 14.5 | 17.6 | heat shock protein |
| stu0120 | O | *DnaK* | 17.3 | 19.8 | molecular chaperone |
| stu0121 | O | *DnaJ* | 10.5 | 11.6 | chaperone protein |
| stu0203 | O | *GroES* | 8.1 | 10.5 | co-chaperonin |
| stu0204 | O | *GroEL* | 5.4 | 5.3 | chaperonin |
| stu0715 | O | *htpX* | 1.8 | 2.1 | heat shock protein |
| stu1614 | O | *ClpL* | 3.2 | 2.7 | ATP-dependent Clp protease |
| stu1823 | O | *radA* | -1.8 | -1.3 | DNA repair protein |
| stu1885 | O | *pepO* | -2.8 | -1.8 | endopeptidase O |
| stu0356 | O | *clpP* | 1.9 | 3.2 | ATP-dependent Clp protease |
| stu0569 | M | *-* | 4.5 | 4.3 | glycosyl transferase family protein |
| stu0570 | M | *-* | 1.4 | 1.8 | glycosyl transferase family protein |
| stu0731 | M | *murD* | 1.6 | 1.8 | UDP-N-acetylmuramoylalanine-D-glutamate ligase |
| stu0732 | M | *murG* | 2.8 | 2.6 | undecaprenyldiphospho-muramoylpentapeptide beta-N-acetylglucosaminyltransferase |
| stu0762 | M | *dltB* | -2.8 | -3.8 | integral membrane protein |
| stu0763 | M | *dltC* | -1.6 | -2.6 | D-alanine-poly(phosphoribitol) ligase |
| stu0764 | M | *dltD* | -2.3 | -2.4 | D-alanine transfer protein |
| stu1607 | M | *-* | -2.6 | -2.2 | hypothetical protein |
| stu0589 | L | *parC* | 2.5 | 2.3 | DNA topoisomerase IV subunit A |
| stu0658 | L | *-* | -1.6 | -1.2 | hypothetical protein |
| stu1209 | L | *hstH* | -2.2 | -1.2 | histone-like DNA-binding protein |
| stu1465 | L | *radC* | 3.1 | 2.5 | DNA repair protein |
| stu0065 | K | *MarR* | 3.8 | 5.2 | transcriptional regulator |
| stu0076 | K | *CtsR* | -4.3 | -4.8 | transcriptional regulator |
| stu0118 | K | *hrcA* | 6.8 | 4.6 | heat-inducible transcription repressor |
| stu0133 | K | *rpoE* | -1.6 | -2.6 | DNA-directed RNA polymerase |
| stu0432 | K | *MarR* | 1.8 | 3.1 | transcriptional regulator |
| stu0452 | K | *LysR* | 2.7 | -1.9 | transcriptional regulator |
| stu0931 | K | *TetR* | -1.6 | 3.3 | transcriptional regulator |
| stu1600 | K | *MerR* | -1.5 | -4.3 | transcriptional regulator |
| stu1868 | K | *rpoB* | -3.8 | -3.4 | DNA-directed RNA polymerase |
| stu0073 | J | *rpsB* | -2.3 | -1.5 | 30S ribosomal protein S2 |
| stu0074 | J | *tsf* | 3.6 | 3.1 | elongation factor Ts |
| stu0093 | J | *rplM* | 1.8 | 2.3 | 50S ribosomal protein L13 |
| stu0094 | J | *rpsI* | 4.2 | 2.3 | 30S ribosomal protein S9 |
| stu0151 | J | *def* | -3.4 | -3.5 | peptide deformylase |
| stu0154 | J | *rpsO* | -5.3 | -5 | 30S ribosomal protein S15 |
| stu0329 | J | *serS* | 1.2 | 1.6 | seryl-tRNA synthetase |
| stu0417 | J | *rplU* | -2.4 | -1.5 | 50S ribosomal protein L21 |
| stu0419 | J | *-* | 5.4 | 4.5 | acetyltransferase |
| stu0451 | J | *metG* | 3.2 | 4.3 | methionyl-tRNA synthetase |
| stu0477 | J | *valS* | -1.6 | -2.5 | valyl-tRNA synthetase |
| stu0572 | J | *thrS* | 1.2 | 1.6 | threonyl-tRNA synthetase |
| stu0746 | J | *rpmE2* | -2.5 | -1.6 | 50S ribosomal protein L31 |
| stu0754 | J | *-* | -2.8 | -3 | SUA5/YciO/YrdC family protein |
| stu0798 | J | *rpsT* | -3.2 | -2.5 | 30S ribosomal protein S20 |
| stu1134 | J | *infC* | -1.8 | -1.6 | translation initiation factor IF-3 |
| stu1490 | J | *rpsU* | 1.9 | 2.3 | 30S ribosomal protein S21 |
| stu1808 | J | *rpmH* | 3.1 | 2.8 | 50S ribosomal protein L34 |
| stu1811 | J | *rnpA* | 2.5 | 1.7 | ribonuclease P |
| stu1814 | J | *gltX* | -3.6 | -3.1 | glutamyl-tRNA synthetase |
| stu1912 | J | *infA* | -2.6 | -1.8 | translation initiation factor IF-1 |
| stu1925 | J | *rpsQ* | 2.6 | 1.7 | 30S ribosomal protein S17 |
| stu1926 | J | *rpmC* | -3.3 | -3.6 | 50S ribosomal protein L29 |
| stu1931 | J | *rplB* | -1.2 | -1.9 | 50S ribosomal protein L2 |
| stu1932 | J | *rplW* | -2.6 | 3.6 | 50S ribosomal protein L23 |
| stu0468 | G | *-* | -2.3 | -2.2 | hypothetical protein |
| stu0488 | G | *tpiA* | 2.6 | 2.5 | triosephosphate isomerase |
| stu1120 | G | *deoB* | -1.6 | -1.8 | phosphopentomutase |
| stu1196 | G | *pyk* | -1.8 | -4.2 | pyruvate kinase |
| stu1671 | G | *gla* | -1.6 | -1.3 | glycerol facilitator |
| stu0030 | F | *purC* | -1.8 | -1.8 | phosphoribosylaminoimidazole-succinocarboxamide synthase |
| stu0032 | F | *purF* | -1.1 | -1.6 | amidophosphoribosyltransferase |
| stu0033 | F | *purM* | -1.8 | -1.5 | phosphoribosylaminoimidazole synthetase |
| stu0807 | F | *cdd* | 2.4 | 3.8 | cytidine deaminase |
| stu1871 | EH | *ilvC* | 2.1 | 2.6 | ketol-acid reductoisomerase |
| stu0158 | E | *-* | -2.5 | -2.6 | polar amino acid transport ATP-binding protein |
| stu0159 | E | *-* | -3.6 | -3.1 | polar amino acid transport substrate-binding protein |
| stu0359 | E | *livJ* | 1.8 | 2.3 | branched-chain amino acid transport substrate-binding protein |
| stu0360 | E | *livH* | 2.3 | 2.2 | branched-chain amino acid transport permease protein |
| stu0361 | E | *livM* | 2.5 | 1.2 | branched-chain amino acid transport permease protein |
| stu0362 | E | *livG* | 1.3 | 1.6 | branched-chain amino acid transport ATP-binding protein |
| stu0363 | E | *livF* | 1.8 | 3.8 | branched-chain amino acid transport system ATP-binding protein |
| stu0646 | E | *aroK* | -2.3 | -1.5 | shikimate kinase |
| stu0846 | E | *cysM2* | -1.6 | -1.7 | cysteine synthase |
| stu0847 | E | *metB2* | -1.8 | -2.5 | cystathionine beta-lyase |
| stu1316 | E | *sdaB* | 4.9 | 2.5 | L-serine dehydratase beta subunit |
| stu1317 | E | *sdaA* | 1.6 | 1.1 | L-serine dehydratase alpha subunit |
| stu1461 | E | *nifS3* | -5 | -3.5 | Putative cysteine desulfurase |
| stu1878 | E | *thrC* | 3.4 | 3 | threonine synthase |
| stu0705 | V | *hsdR1* | 4.5 | 4.6 | type I restriction-modification system |
| stu0708 | V | *hsdS1* | 1.8 | 1.7 | type I restriction-modification system |
| stu0711 | V | *hsdM1* | 2.3 | 2.1 | type I restriction-modification system |
| stu0317 | TK | *CovR* | 1.8 | 4.2 | response regulator |
| stu1380 | TK | *-* | -1.8 | -1.7 | response regulator |
| stu1420 | TK | *rr08* | 2.3 | 1.9 | response regulator |
| stu0318 | T | *CovS* | 2.1 | 2.2 | sensor histidine kinase |
| stu1421 | T | *hk08* | 1.8 | 2.5 | sensor histidine kinase |
| stu0003 | S | *-* | 1.1 | 2.3 | hypothetical protein |
| stu0075 | S | *-* | 1 | 1.8 | hypothetical protein |
| stu0090 | S | *-* | 1.6 | 1.5 | hypothetical protein |
| stu0423 | S | *-* | -3.3 | -2.7 | hypothetical protein |
| stu0440 | S | *-* | 2.8 | 4.6 | hypothetical protein |
| stu0508 | S | *-* | -1.5 | -1.9 | hypothetical protein |
| stu0657 | S | *-* | -1.8 | -2.1 | hypothetical protein |
| stu0659 | S | *-* | -2.3 | -2.5 | hypothetical protein |
| stu0815 | S | *-* | 1.8 | 1.2 | hypothetical protein |
| stu0830 | S | *-* | 3 | 1.6 | hypothetical protein |
| stu0832 | S | *-* | 2.3 | 2 | hypothetical protein |
| stu0833 | S | *-* | 1.6 | 1.7 | hypothetical protein |
| stu0868 | S | *-* | -9.3 | -5.4 | hypothetical protein |
| stu0888 | S | *-* | -8.0 | -5.6 | hypothetical protein |
| stu0998 | S | *-* | 3.2 | 1.6 | hypothetical protein |
| stu1118 | S | *-* | -1.7 | -1.3 | hypothetical protein |
| stu1248 | S | *-* | 1.8 | 3.5 | hypothetical protein |
| stu1253 | S | *-* | -4.1 | -3.9 | hypothetical protein |
| stu1285 | S | *-* | 1.6 | 1.1 | hypothetical protein |
| stu1459 | S | *-* | 3.6 | 2 | hypothetical protein |
| stu1866 | S | *-* | 1.2 | 1.6 | hypothetical protein |
| stu1959 | S | *-* | -2 | -1.7 | hypothetical protein |
| stu1993 | S | *-* | -1.5 | -1.6 | putative membrane protein |
| stu1996 | S | *-* | -1.8 | -3 | putative membrane protein |
| stu2011 | S | *-* | 1.9 | 1.4 | hypothetical protein |
| stu0089 | R | *-* | -2.4 | -2.8 | hypothetical protein |
| stu0831 | R | *-* | 1.8 | 2.3 | hypothetical protein |
| stu1147 | R | *-* | 1.6 | -1.3 | hypothetical protein |
| stu1249 | R | *-* | -2.5 | -2.3 | hypothetical protein |
| stu1487 | R | *-* | -1.1 | 2.5 | hypothetical protein |
| stu1615 | R | *-* | -3.5 | -3.7 | hypothetical protein |
| stu1665 | R | *-* | -3.3 | -2.1 | putative ABC transporter permease protein |
| stu1666 | R | *-* | -2.7 | -3.3 | putative ABC transporter ATP binding protein |
| stu1802 | R | *-* | 7.6 | 4.6 | hypothetical protein |
| stu1847 | R | *-* | -2 | -2.3 | hypothetical protein |
| stu1875 | R | *-* | 2.8 | 4.3 | hypothetical protein |
| stu0607 | P | *feoA* | 2.7 | 3.5 | ferrous iron uptake transporter protein A |
| stu0608 | P | *feoB* | 1.8 | 2.1 | ferrous iron uptake transporter protein B |
| stu0723 | P | *dpr* | -2.6 | -2.1 | peroxide resistance protein |
| stu0724 | P | *Fur* | -3 | -3.4 | transcriptional regulator |
| stu1001 | P | *pstS* | 1.6 | 1.8 | phosphate ABC uptake transporter substrate binding protein |
| stu1002 | P | *pstC1* | 4.5 | 3.2 | phosphate transport system permease protein |
| stu1003 | P | *pstC2* | 3.8 | 2 | phosphate transport system permease protein |
| stu1004 | P | *pstB1* | 1.6 | 1.4 | phosphate transport system ATP-binding protein |
| stu1005 | P | *pstB2* | 5.3 | 5.6 | phosphate transport system ATP-binding protein |
| stu1006 | P | *phoU* | 2.3 | 1.7 | phosphate uptake regulatory protein |
| stu1024 | P | *-* | -2.4 | -2.2 | putative lipoprotein |
| stu1025 | P | *fatB* | 2.4 | 3.1 | iron complex transport substrate-binding protein |
| stu1026 | P | *fatA* | 1.2 | 2 | iron complex transport ATP-binding protein |
| stu1027 | P | *fatC* | 1.6 | 1.2 | iron complex transport permease protein |
| stu1028 | P | *fatD* | 2.5 | 1.8 | iron complex transport permease protein |
| stu1416 | D | *ftsK* | -2.9 | -2.2 | cell division protein |
| stu0478 | C | *atpE* | -6.3 | -4.8 | F0F1 ATP synthase |
| stu0479 | C | *atpB* | -3.2 | -3.3 | F0F1 ATP synthase |
| stu0485 | C | *atpC* | -2 | -1.9 | F0F1 ATP synthase subunit epsilon |
| stu0557 | C | *-* | -2.3 | -2.5 | pyridine nucleotide-disulfide oxidoreductase |
| stu1137 | C | *fer* | -1.9 | -1.8 | ferredoxin |
| stu1369 | C | *-* | 2.8 | 1.6 | NAD(P)H nitroreductase |
| stu0013 | *-* | *-* | -3.2 | -3.7 | hypothetical protein |
| stu0036 | *-* | *-* | 1.7 | 3.2 | hypothetical protein |
| stu0087 | *-* | *-* | 3 | 1.6 | hypothetical protein |
| stu0106 | *-* | *-* | -1.5 | -2.4 | hypothetical protein |
| stu0135 | *-* | *-* | -3.4 | -5.4 | hypothetical protein |
| stu0161 | *-* | *-* | 2.4 | 3.6 | hypothetical protein |
| stu0179 | *-* | *-* | -1.7 | 3.2 | hypothetical protein |
| stu0221 | *-* | *-* | 1.5 | 1.7 | hypothetical protein |
| stu0293 | *-* | *-* | 1.8 | 1.6 | hypothetical protein |
| stu0354 | *-* | *-* | 1.3 | 2.5 | hypothetical protein |
| stu0473 | *-* | *-* | -1.4 | 2.8 | hypothetical protein |
| stu0475 | *-* | *-* | 1.8 | 1.6 | hypothetical protein |
| stu0516 | *-* | *-* | -1.4 | -2.4 | hypothetical protein |
| stu0565 | *-* | *-* | -2.7 | -3.1 | hypothetical protein |
| stu0574 | *-* | *-* | 2.5 | 2.4 | hypothetical protein |
| stu0580 | *-* | *-* | -1.6 | -2 | hypothetical protein |
| stu0591 | *-* | *-* | -2.3 | -1.8 | hypothetical protein |
| stu0610 | *-* | *-* | -3.2 | -2.2 | hypothetical protein |
| stu0651 | *-* | *-* | 2.3 | 1.4 | hypothetical protein |
| stu0695 | *-* | *-* | -2.4 | -2.5 | hypothetical protein |
| stu0756 | *-* | *-* | -2.3 | 1.2 | hypothetical protein |
| stu0829 | *-* | *-* | 2.8 | 3.3 | hypothetical protein |
| stu0835 | *-* | *-* | 1.6 | 1.5 | hypothetical protein |
| stu0859 | *-* | *-* | -2.6 | -2 | hypothetical protein |
| stu0863 | *-* | *-* | 4 | 3.5 | hypothetical protein |
| stu0944 | *-* | *-* | -3.2 | -2.6 | hypothetical protein |
| stu0992 | *-* | *-* | -1.6 | -1.7 | hypothetical protein |
| stu1047 | *-* | *-* | -4.2 | -3.6 | hypothetical protein |
| stu1075 | *-* | *-* | 1.4 | 3.7 | hypothetical protein |
| stu1168 | *-* | *-* | -2.2 | -2.1 | hypothetical protein |
| stu1191 | *-* | *-* | 2.3 | 1.5 | hypothetical protein |
| stu1238 | *-* | *-* | -3.1 | -2.1 | hypothetical protein |
| stu1320 | *-* | *-* | 2.6 | 2.7 | hypothetical protein |
| stu1360 | *-* | *-* | 2.1 | -1.2 | hypothetical protein |
| stu1378 | *-* | *-* | 3.6 | 2 | hypothetical protein |
| stu1383 | *-* | *-* | 2.2 | 2.6 | hypothetical protein |
| stu1454 | *-* | *-* | -3.5 | -1.6 | hypothetical protein |
| stu1533 | *-* | *-* | -3.6 | -3 | hypothetical protein |
| stu1575 | *-* | *-* | 4 | 1.6 | hypothetical protein |
| stu1639 | *-* | *-* | -3.9 | -1.9 | hypothetical protein |
| stu1692 | *-* | *-* | -1.7 | -1.2 | hypothetical protein |
| stu1722 | *-* | *-* | 2.8 | 3.4 | hypothetical protein |
| stu1764 | *-* | *-* | -1.5 | 3.1 | hypothetical protein |
| stu1783 | *-* | *-* | -2 | -1.6 | hypothetical protein |
| stu1853 | *-* | *-* | 1.6 | 1.8 | hypothetical protein |
| stu1855 | *-* | *-* | -3.1 | -1.9 | hypothetical protein |
| stu1938 | *-* | *-* | 1.2 | 1.6 | hypothetical protein |
| stu1981 | *-* | *-* | -1.8 | -2.7 | hypothetical protein |
| stu1994 | *-* | *-* | -2.2 | -3 | hypothetical protein |
